# MODEL‐AD Pre‐Clinical Testing Core: A Precision Medicine Pipeline for Preclinical Testing of Alzheimer’s Disease Therapeutic Candidates

**DOI:** 10.1002/alz.086531

**Published:** 2025-01-03

**Authors:** Paul R Territo

**Affiliations:** ^1^ Indiana University, Indianapolis, IN USA

## Abstract

**Background:**

Preclinical testing in animal models is a critical component of the drug discovery process. Over the past three decades hundreds of interventions have demonstrated preclinical efficacy for ameliorating cognitive impairments in animal models; however, none have translated to efficacy in Alzheimer’s disease (AD) clinical trials. This lack of translation suggests that there are issues with the animal models employed, the preclinical assays, and poor scientific rigor and reproducibility during execution. To overcome these limitations, the Preclinical Testing Core (PTC) of the Model Organism Development and Evaluation for Late‐onset AD (MODEL‐AD) consortium has established a rigorous screening strategy with go/no‐go decision points that permits unbiased assessments of therapeutic agents.

**Method:**

For each compound selected for screening, a mouse model that is best matched to the drugs mechanism of action, and that has been comprehensively characterized by the MODEL‐AD consortium, will be enrolled. An initial screen evaluates drug identity, stability, formulation, pharmacokinetics (PK), and simulations to confirm appreciable brain exposure in the disease model at the pathologically relevant ages. These efforts inform on the dose regimen for long‐term studies, where secondary pharmacodynamics studies (PD) evaluates target engagement utilizing non‐invasive PET/CT and post‐mortem confirmation. Provided the compound meets the “go” criteria for these endpoints, evaluation for behavioral endpoints are conducted as well as post treatment transcriptomic and proteomic analyses.

**Result:**

Validation of this pipeline using both small molecule and biologic tool compounds including the evaluation of verubecestat, levetiracetam, and aducanumab revealed the importance of critical quality control (QC) steps when executing preclinical studies. These include confirmation of the active pharmaceutical ingredient, PK, and PD in well powered studies powered to evaluate effects of sex and conducted according to the ARRIVE guidelines.

**Conclusion:**

Based on our experience executing rigorous screening strategies with QC checkpoints provide insight to the challenges of conducting translational studies in animal models. The PTC pipeline, an NIA resource, is accessible to the research community for investigators to nominate compounds for testing (https://stopadportal.synapse.org/), thus enabling unbiased studies that support clinical translation.

